# Swimming exercise attenuates LPS-induced spatial memory deficits associated with IL-17/NF-κB pathway and synaptic dysfunction in developing mice

**DOI:** 10.3389/fimmu.2026.1954022

**Published:** 2026-10-28

**Authors:** Lulu Shi, Weiting Li, Su Zhan, Xiaocheng Liu, Zhuhong Wu, Zhibin Liu, Chong Chen, Songhua Wang, Jiang-Hua Li

**Affiliations:** 1 Physical Education College, Jiangxi Normal University, Nanchang, China; 2 School of Sports and Health, Jiangxi Science and Technology Normal University, Nanchang, China; 3 Jiangxi Provincial Key Laboratory of Organic Functional Molecules, Institute of Organic Chemistry, Jiangxi Science and Technology Normal University, Nanchang, China; 4 Key Laboratory of Infection and Immunity, Health Commission of Jiangxi Province, Nanchang and School of Basic Medicine, Nanchang Medical College, Nanchang, China

**Keywords:** LPS, NF-κB pathway, spatial memory, swimming, synaptic activity

## Abstract

**Background:**

Aerobic exercise is known to enhance learning and memory capabilities, whereas inflammation is detrimental to these cognitive functions. This study aimed to investigate the potential of swimming exercise to mitigate spatial memory deficits induced by lipopolysaccharide (LPS) in developing mice.

**Results:**

Swimming mitigated spatial memory deficits in LPS-treated mice. Transcriptomic analysis indicated that hippocampal gene expression profiles were significantly associated with inflammatory responses, particularly implicating the IL-17/NF-κB signaling pathway. Further investigation using western blotting demonstrated that LPS administration increased the expression of hippocampal inflammatory factors (IL-6 and IL-17) and components of NF-κB p65, while decreasing synaptic proteins (GluA1). In contrast, swimming exercise reduced hippocampal inflammatory factors and NF-κB pathway components and increased synaptic proteins. Additionally, LPS decreased both the frequency and amplitude of spontaneous excitatory postsynaptic currents (sEPSCs) in the CA1 region, as well as the amplitude of evoked excitatory postsynaptic currents (evoked EPSCs) and long-term potentiation (LTP) in the Schaffer collateral-CA1 pathway. Conversely, swimming exercise restored the frequency and amplitude of sEPSCs in the CA1 region and enhanced the amplitude of EPSCs. In conclusion, swimming alleviates LPS-induced spatial memory deficits in juvenile mice, an effect associated with modulation of the IL-17/NF-κB pathway and the enhancement of synaptic activity.

## 1 Introduction

Spatial memory refers to the cognitive ability to acquire, retain, and use information related to the topographical organization of environments. This includes the recognition of objects and landmarks, as well as the navigation between distinct locations​ (1). The hippocampus, encompassing the CA1, CA3, and dentate gyrus (DG) subregions, is critically implicated in the regulation of spatial memory (2–6). Neuroinflammation is significantly associated with the pathogenesis of neurodegenerative disorders, such as mild cognitive impairment (7). Lipopolysaccharide (LPS), an endotoxin derived from the outer membrane of gram-negative bacteria (8), acts as a potent immunological and inflammatory stimulus widely employed in studies of both the central nervous system and peripheral tissues. LPS administration induces neuroinflammatory responses within the hippocampus and promotes neuronal apoptosis, ultimately leading to memory impairment. This effect is mediated primarily through the activation of the nuclear factor kappa B (NF-κB) signaling pathway and upstream inflammatory factors, including interleukin-1β (IL-1β), interleukin-6 (IL-6), and tumor necrosis factor-α (TNF-α). This pathway involves the key signaling molecules NF-κB p65, IκBα, and IKKα (9–12). Importantly, emerging evidence has identified interleukin-17 (IL-17) as a critical upstream mediator that not only amplifies NF-κB activation but also directly regulates hippocampal synaptic plasticity. IL-17 signaling through its receptor IL-17RA has been shown to disrupt long-term potentiation (LTP) in the hippocampal CA1 region via p38 MAPK activation (13), and IL-17 deficiency results in impaired synaptic plasticity and memory deficits (14). Recent experimental evidence indicates that intraperitoneal injection of LPS (0.1–0.5 mg/kg) induces deficits in spatial learning and memory, concomitant with alterations in neuroprotective biomarkers associated with memory function (7, 10, 15, 16).

Physical activity contributes to the prevention of cognitive decline and neurodegenerative diseases (17, 18). Research has corroborated that aerobic exercise exerts a significant neuroprotective effect in delaying cognitive decline (19). Twenty-four weeks of low-intensity and high-intensity exercise can enhance cognitive function in patients with senile dementia (20). Voluntary exercise during the developmental stage can augment spatial memory in mice by increasing the number of synapses and improving synaptic transmission in the hippocampus (6). Four-week treadmill exercise can mitigate LPS-induced neuroinflammation and improve spatial memory levels in adult mice (7). Other research has shown that swimming training is associated with a reduction in memory impairment, IL-6, IL-1β, and TNF-α in the hippocampus and prefrontal cortex of type 2 diabetic mice (21). Among various exercise modalities, swimming has emerged as a particularly advantageous model for studying the neuroprotective effects of aerobic exercise. Unlike treadmill running, which requires electric shock stimulation and imposes forced confinement that may elevate stress hormones and confound experimental outcomes, swimming is an inherent locomotor behavior in rodents that elicits minimal stress responses (22). Moreover, swimming necessitates less elaborate equipment compared to treadmill or voluntary wheel exercise, and group swimming has been shown to significantly increase exercise intensity through social interaction among animals (23). Importantly, swimming training has been demonstrated to reduce hippocampal levels of IL-6, IL-1β, and TNF-α in various pathological models (21), and recent studies have shown that swimming promotes skeletal muscle-derived extracellular vesicles that cross the blood-brain barrier to enhance microglial clearance of pathological proteins (24). Moreover, Elgizawy et al. recently reported neuroprotective and anti-inflammatory effects of lifestyle-related interventions in sleep-deprived rats, investigating inflammatory markers and CLOCK regulation, which provides useful comparative evidence for the broader anti-inflammatory and neuroprotective effects of exercise-related interventions (25). Given that swimming is a safe, accessible, and child-friendly form of aerobic exercise, it holds particular translational relevance for pediatric populations. In addition, the neuroimmune mechanisms underlying neurodevelopmental disorders reveal that the production of IL-17a during inflammation can improve the manifestation of social behavior deficits by directly influencing neuronal activity in the central nervous system (26).

Early-stage inflammation results in cognitive decline in children (27, 28). Despite growing evidence linking neuroinflammation to cognitive impairment, several critical knowledge gaps remain. First, it is unclear whether swimming exercise, as a stress-free aerobic intervention, can ameliorate LPS-induced spatial memory deficits specifically through modulation of the IL-17/NF-κB signaling axis during brain development. Second, while the NF-κB pathway has been extensively studied in the context of exercise and cognition, the role of IL-17—a cytokine that sits at the intersection of adaptive immunity and neuroinflammation—remains poorly understood in developing brains. Third, our preliminary transcriptomic analysis (RNA-seq combined with KEGG enrichment) revealed that hippocampal gene expression profiles were significantly correlated with inflammatory responses, notably implicating the IL-17/NF-κB signaling pathway as a key target of swimming intervention. However, whether swimming exercise exerts its cognitive-protective effects by directly targeting this pathway has not been experimentally verified. Can swimming training during the developmental stage improve the cognitive function of male mice exposed to LPS? This study explores the influence of swimming on LPS-induced spatial memory impairments, which are intricately associated with synaptic function and the alleviation of neuroinflammation in developing mice. The objective was to clarify the potential effect of swimming on the spatial memory ability of developing mice and further elucidate its underlying neural mechanisms, with a specific focus on the IL-17/NF-κB signaling pathway and its downstream effects on synaptic function.

## 2 Materials and methods

All work described in this study was approved by the Institutional Animal Care and Use Committee of Jiangxi Science and Technology Normal University (3601020137931). All animal experiments complied with the ARRIVE guidelines. All mice were housed under standard laboratory conditions with a 12 h light/dark cycle. The ambient temperature was maintained at 22 ± 2 °C, and the relative humidity was maintained at 50% ± 5%. All animals had free access to standard chow and drinking water. The animals were randomly allocated to experimental groups, with each assigned a unique identification number. Random numbers were generated using the RAND function in Microsoft Excel to facilitate the group allocation. The investigators were blinded to the group identities during data acquisition and analysis.

### 2.1 Experimental animals and swimming exercise

Four-week-old weight-matched C57BL/6J male mice (N = 54) were selected. Six mice were housed per cage. They were randomly divided into the control (N=18), LPS (N = 18), and swim + LPS groups (N=18). As an inherent ability of rodents, swimming exercise offers advantages compared to treadmill running. Additionally, swimming necessitates a less elaborate device in comparison to treadmill running and spontaneous wheel exercise (22). Swimming training was adopted in the swim and swim + LPS groups. The animals were swum as a group of six mice, because it has been demonstrated that the intensity of swimming exercise was significantly raised by the interaction among the animals (23). The specific plan was as follows: The mice underwent training in a water tank (with dimensions of 100 cm in length, 60 cm in width, and 80 cm in height) to swim without additional weight, and the water temperature was maintained at 32˚C with a tolerance of ±1˚C. The water depth was set at 60 cm to prevent the mice from supporting themselves by touching the bottom with their feet. The swimming training was divided into an adaptation and a training period. The adaptation period lasted for 1 week in the fifth week. During the initial five-day period, the mice swam for 5 minutes, 10 minutes, 15 minutes, 20 minutes, and 30 minutes respectively, followed by a two - day rest period. Subsequently, the training phase endured for four weeks. The mice swam five days per week, with each swimming session lasting 30 minutes. Considering that the mice were in their juvenile stage and in a swimming state, 30 minutes was selected as the training duration in the sixth to ninth week. Swimming exercises were performed at the same time of day (between 9:00 AM and 11:00 AM) to minimize circadian rhythm-related variability. After swimming, the mice were dried using a towel and returned to their cages. Throughout the swimming training, daily records of the mice’s body weight changes were maintained. During the nineth week, the LPS group was administered intraperitoneal injections of 0.1 mg/kg LPS once daily, while the control group was administered an equivalent volume of saline (sterile 0.9% NaCl). In the tenth week, we conducted the behavioral test and carried out tissue collection, some mice were subjected to electrophysiological experiments, and the remaining mice were sacrificed. Hippocampal samples were collected and immediately stored at – 80 ^°^C for subsequent western blot analyses. **Figure 1A** presents a summary of the experimental design.

**Figure 1:**
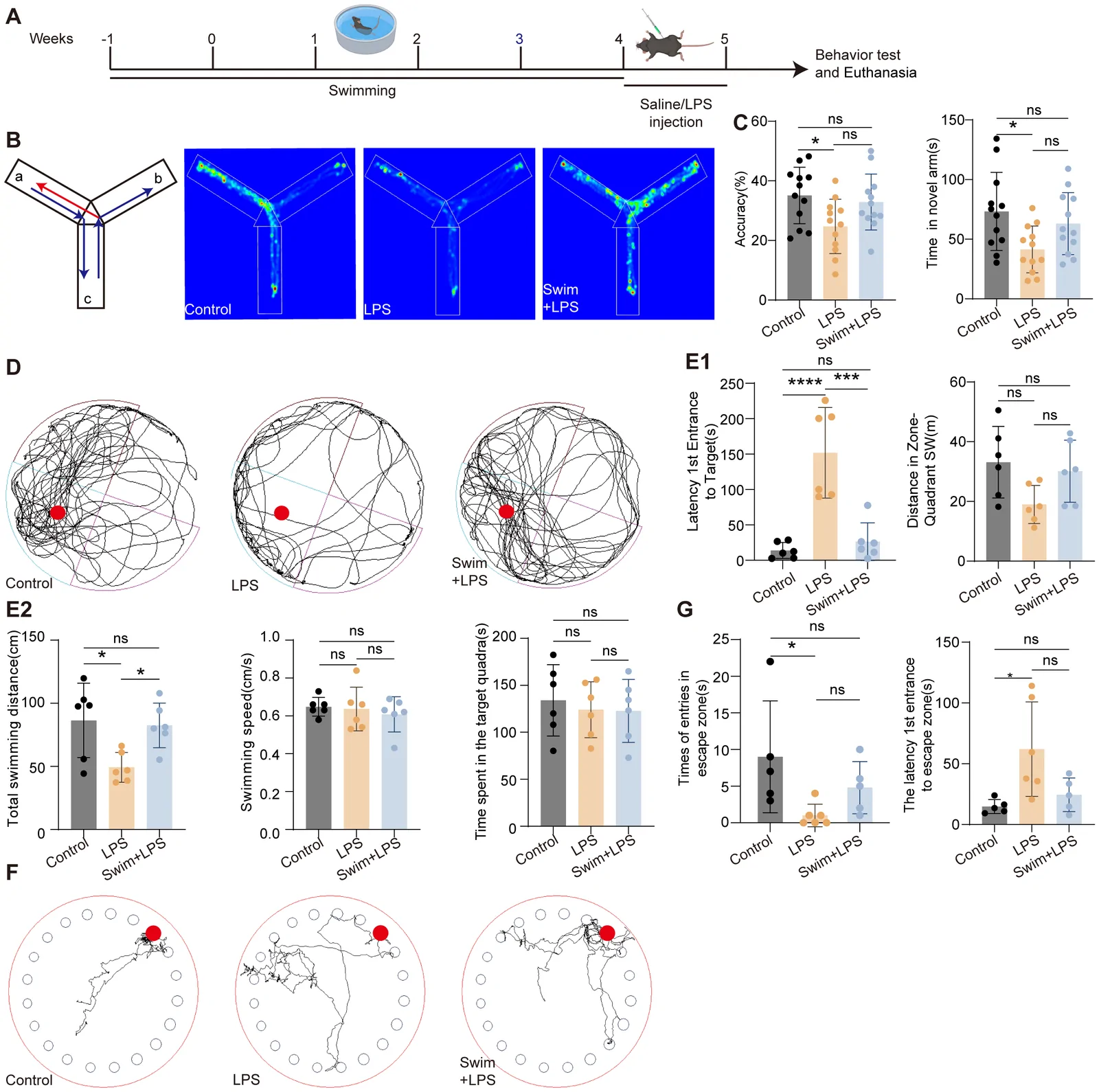
Swimming exercise ameliorates lipopolysaccharide-induced decline in spatial memory. **(A)** Diagrammatic representation of the experimental procedures. **(B)** The Y-maze test demonstrated the behavioral patterns of mice, with the red arrow indicating a familiar pattern and blue lines denoting a novel pattern (left). The movement trajectories of the three groups of mice are illustrated using heat maps (right panel). **(C)** Statistical representation of arm entry accuracy (left) and time spent in the novel arm (right), n=12 for each group. **(D)** Trajectory map of mice in the water maze, n=12 for each group. **(E1)** Latency to the first entrance to the target (left) and distance in the zone quadrant SW (right)statistical analysis chart, n=6 for each group. **(E2)** Total swimming distance(left), swimming speed(middle) and time spent in the target quadrant statistical analysis chart, n=6 for each group. **(F)** Trajectory map of mice in the Barnes Maze. **(G)** Time of entries into the escape zone (left, n=5, 6, 5 for Control, LPS, and swimming+LPS group, respectively) and latency to first escape from the zone (right, n=5, 6, 5 for Control, LPS, and swimming+LPS group, respectively) statistical analysis chart. Unpaired t-test analysis, *p < 0.05, ***p < 0.001, ****p < 0.0001.

### 2.2 Behavioral analysis

To clarify the experimental design, mice from each group (n = 18) were allocated into two independent cohorts. Cohort A (n = 12 per group) underwent Y-maze testing, comprising the spontaneous alternation experiment followed by the novel arm exploration experiment with a 1-h interval. Cohort B (n = 6 per group) was used for both the Morris Water Maze (MWM) and Barnes maze tests. The testing sequence was: Y-maze (Cohort A) first, followed by MWM (Cohort B), and finally Barnes maze (Cohort B) after a 3-day recovery interval following MWM completion. All behavioral tests were performed between 12:00 PM and 4:00 PM to control for circadian influences.

To minimize potential confounding effects of locomotor activity, motivation, fatigue, and stress on behavioral outcomes, several control measures were implemented throughout the experimental protocol. First, regarding locomotor activity, swimming speed was recorded during the MWM test to verify that intergroup differences in spatial memory performance were not attributable to differences in swimming ability; indeed, no significant differences in swimming speed were observed among the three groups (Control: 0.65 ± 0.05 m/s; LPS: 0.64 ± 0.12 m/s; Swim+LPS: 0.61 ± 0.09 m/s). Second, to standardize motivation across groups, all animals received identical handling procedures; the Barnes maze employed uniform aversive stimuli (white noise at 85 dB and bright light at 300 lx), the MWM leveraged the innate motivation to escape water, and the Y-maze assessed spontaneous alternation driven by innate exploratory curiosity. Third, to prevent fatigue, mice underwent a 1-week adaptation period with progressively increasing swim durations (5, 10, 15, 20, and 30 min), followed by a 2-day rest period before the training phase; daily body weight changes were monitored as an indicator of overall health and fatigue; and the 30-min daily swim duration was selected considering the juvenile developmental stage. Fourth, to minimize stress, swimming was selected as the exercise modality because it is an innate rodent behavior that elicits minimal stress responses compared to forced treadmill running, which requires electric shock stimulation (22); additionally, all animals were housed under standardized conditions (12 h light/dark cycle, 22 ± 2 °C, 50% ± 5% humidity) with ad libitum access to food and water, and behavioral testing was conducted during the active (dark) phase.

#### 2.2.1 Y-maze tests

The Y-maze test (every group: N = 12) is widely employed to evaluate hippocampus-dependent memory functions, encompassing both working memory and reference memory (29, 30). Specifically, the spontaneous alternation paradigm has been applied to assess spatial working memory, whereas the novel arm exploration paradigm, which incorporates a delay interval between familiarization and test phases, is used to evaluate spatial reference memory (29, 31–33).

Behavioral tests were conducted using a Y-maze (RWD, Shenzhen, China) consisting of three equal arms and a central intersection area fabricated from medical plexiglass. Each arm of the Y-maze was 30 cm long, 8 cm wide, and 15 cm high, with an angle of 120° between any two adjacent arms. The side walls and bottom of the maze were black, whereas the top was covered with a transparent material to enable the camera positioned above the maze to clearly record the behavior of the experimental animals.

#### 2.2.2 Spontaneous alternation experiments

The spontaneous alternation experiment serves as a methodology for evaluating spatial working memory. 12 mice were randomly selected from each group to participate in this test. Upon being placed in the Y-maze, the working memory of the test mouse enabled it to recollect which arm had been previously explored. Driven by their inherent curiosity, mice within the maze exhibit a tendency to explore arms that have not been recently accessed. The entry of all four paws of the mouse into another arm of the Y-maze can be regarded as entering a new arm. During the test, the activity trajectory of the test mouse was recorded for 8 minutes immediately after it was introduced into the Y-maze, and the total number (N) and sequence of entries into the three arms of the maze were analyzed. A consecutive entry into three different arms was counted as a correct instance, and the ratio of the correct number (n) to N-2 represents the spontaneous alternating correct rate (34). After each trial, the test mouse was removed from the maze, and the inner walls and floor were disinfected with alcohol to eliminate any residual odor.

#### 2.2.3 Novel arm exploration experiments

Novel arm exploration experiments were conducted to assess spatial reference memory, with 12 mice in each group undergoing the tests. During the testing process, one arm of the Y-maze was randomly selected as the novel arm and separated from other areas by a baffle to preclude temporary access. Prior to the test, a mouse was positioned in an undesignated arm and provided with a 15-minute interval for unrestricted exploration of its surrounding milieu. After this interval, the mice were returned to their original positions. One hour later, the isolation barrier was removed, and the same mouse re-entered the maze through the starting point. The subjects’ activities in all three arms of the Y-maze were recorded for 5-minute using a camera. Subsequently, the mouse was removed from the maze, and both the inner walls and underside of the maze were disinfected with alcohol. The frequency ratio, calculated as the number of entries into the novel arm divided by the total number of entries into all arms, and the time ratio, determined as the time spent in the novel arm divided by the total time spent in all arms, were utilized to evaluate the spatial reference memory capacity. A higher value of both ratios implies stronger spatial reference memory (35).

#### 2.2.4 Morris water maze test

The Morris Water Maze (MWM) was used to evaluate the learning and memory capacities of mice (10). The MWM was a circular pool with a diameter of 120 cm and a depth of 50 cm filled with water opacified with non-toxic white ink to a depth of 30 cm, and the water temperature was maintained at 22 ± 1 °C throughout the experiment. A circular escape platform (10 cm in diameter) was submerged 1 cm below the water surface and fixed in the center of one quadrant (target quadrant) during the acquisition phase. The MWM comprised two experiments, and the specific experimental protocols were as follows (36): (Each group: N = 6).

For the orientation navigation experiment, the mice were trained over a four-day period. After training, the researchers recorded the time (within 90 s) taken by a mouse to find the hidden platform, which was referred to as the latency to first entry into the target quadrant (practice experiment). If the mouse failed to locate the platform within 90 s, it was guided to the platform for a 15 s learning session before the next navigation experiment. This experiment was repeated four times per day.

Subsequently, on the fifth day, a test of spatial exploration experiment was conducted. The escape platform was removed, and the mice were placed into the water facing the pool wall from the quadrant opposite to the original escape platform location. The latency to the first entry into the target quadrant, the distance traveled within the target quadrant, swimming speed, total swimming distance, and time spent in the target quadrant of the mice within 300 s were documented.

#### 2.2.5 Barnes maze testing

The Barnes maze is a task used to assess spatial learning and memory in rodents (37). The Barnes maze consists of a circular platform with 18 evenly spaced holes, one of which has a goal box located beneath it. The goal box remained fixed, allowing visual cues to assist the mice during training and testing. To motivate the mice, white noise (85 dB) and bright light (300 lx) were applied during each trial. Surrounding the platform were black geometric cues—crosses, circles, squares, and triangles—positioned 50 cm above the platform at a height of 30 cm. The platform was rotated clockwise between trials, ensuring that any hole could align with the goal box.

Testing was performed using followed methods similar to those described by Barnes (37) (Each group: N = 6). First, the mice were placed in the goal box for 2 min for habituation. They were then positioned at the center of the maze and gently guided to the goal box for another 2 min before being returned to their home cage. After a 15-minute interval, acquisition training began. The mice were placed in a starter cylinder at the center of the maze. After a 30 s delay, the cylinder was removed, and the mice had 4 min to explore the maze and locate the goal box. If the goal box was not found within that time, the mouse was guided there and allowed to remain for 2 min before returning to its home cage.

This procedure was repeated for three additional trials, spaced 15 min apart, and conducted over four days, with four trials conducted each day. Retention testing was conducted three days after the acquisition phase, with mice placed at the center of the maze without the starter cylinder. One trial was conducted each day. Testing occurred between 12:00 PM and 4:00 PM, three hours after the lights were turned off. After each trial, the maze and goal box surfaces were cleaned with a solution of 1.5% ethanol and 1% detergent in water.

Animal behavior analysis was performed by using Y-maze tests, MWM and Barnes maze testing by using a video tracking software [SMART 3.0, The data were analyzed using the SMART 3.0 software (Harvard Apparatus, Holliston, MA, USA)].

### 2.3 RNA sequencing

Brain tissue samples were randomly selected from each group (N = 3 per group), frozen, and subjected to RNA extraction. The purity, quality, and integrity of RNA samples were assessed. mRNA was enriched using oligo(dT)25 magnetic beads, and cDNA libraries were constructed by Wuhan Servicebio Technology Co., Ltd. (Wuhan, China). All nine samples (three biological replicates per group) passed the quality control criteria and were included in the downstream differential expression analysis. In this study, all RNA-seq libraries were prepared [simultaneously/in a single sequencing batch] by [Wuhan Servicebio Technology Co., Ltd.], minimizing the potential for batch effects. [The RNA-seq libraries for all nine samples were sequenced in a single lane/run of the Illumina platform, thereby eliminating inter-batch variability.] [OR: The samples were sequenced across [X] batches. To assess potential batch effects, we performed [PCA analysis/surrogate variable analysis (SVA)], and [no significant batch effects were detected/batch was included as a covariate in the differential expression model].The raw sequencing data (FASTQ files) generated in this study have been deposited in the Science Data Bank, database web is https://www.scidb.cn/anonymous/TW5Zcnlx.

#### 2.3.1 Quality control

Raw sequencing data in the FASTQ format were processed using fastp, an ultra-fast FASTQ preprocessor, to perform quality control. Clean reads were generated after adapter trimming, quality filtering, and per-read quality trimming. All subsequent analyses were conducted using high-quality (38) clean data.

#### 2.3.2 Read mapping to the reference genome

The reference genome and gene model annotation files were obtained from the relevant genome database. Paired-end clean reads were aligned to the reference genome using STAR version 2.7.8 a (39).The paired-end clean reads were aligned to the [Mus musculus] reference genome [GRCm39/mm39/GRCm38/mm10] obtained from [Ensembl/NCBI]. The corresponding gene model annotation file was derived from [Ensembl release X.X/Gencode M28/NCBI Build X.X].

#### 2.3.3 Quantification of gene expression level

StringTie version 2.2.1 was employed to quantify the read counts mapped to each gene. Subsequently, the Transcripts Per Million (TPM) value for each gene was computed based on the gene length and the corresponding read count. Transcripts per kilobase of exon model per million mapped reads (TPM) simultaneously accounts for corrections in sequencing depth and gene length and is widely regarded as the standard method for estimating gene expression levels (40). Sequencing was performed as [paired-end 2×150 bp/single-end 150 bp] sequencing on the [Illumina NovaSeq/HiSeq/NextSeq] platform. After quality control with fastp, the clean data for each sample contained [X.X] million reads with Q20 percentage > [98]% and GC content ranging from [XX]% to [XX]%. The mapping rates to the reference genome using STAR were as follows (**Table 1**):

**Table 1:** Summary of read mapping for RNA sequencing samples.

Group	Sample ID	Total Reads	Mapped Reads	Mapping Rate (%)
Control	Control-1	5,649,6322	5,563,5473	98.4763
Control	Control-2	5,661,2882	5,571,2640	98.4098
Control	Control-3	5,731,7254	5,646,5761	98.5144
LPS	LPS-1	6,437,6638	6,344,5368	98.5534
LPS	LPS-2	4,789,6658	4,720,0441	98.5464
LPS	LPS-3	5,981,6590	5,889,7287	98.4631
Swim+LPS	Swim+LPS-1	4,936,9842	4,852,6706	98.2922
Swim+LPS	Swim+LPS-2	6,063,6038	5,967,6172	98.4170
Swim+LPS	Swim+LPS-3	5,685,0956	5,596,9962	98.4503

#### 2.3.4 KEGG enrichment analysis of differentially expressed genes

Differentially expressed genes (DEGs) were identified using [DESeq2/EdgeR/] based on the [negative binomial distribution model/empirical Bayes statistical framework]. Specifically, the raw read counts obtained from StringTie quantification were imported into [DESeq2/EdgeR], where normalization was performed using [the median-of-ratios method (DESeq2)/TMM normalization (EdgeR)] to account for differences in library size and RNA composition between samples. The statistical model accounted for the experimental design by comparing [specific contrasts, e.g., LPS vs. Control, Swim+LPS vs. LPS] with [three] biological replicates per group. A fold-change threshold of |log2(fold change)| ≥ [1] was applied to define biologically meaningful differential expression. Statistical significance was assessed using the [Benjamini-Hochberg] procedure to control the false discovery rate (FDR). Genes with an adjusted P-value (FDR) < [0.05] were considered statistically significantly differentially expressed. The combination of |log2FC| ≥ [1] and FDR < [0.05] was used as the dual criteria for defining DEGs.STAR version 2.7.8 was used for read alignment, and the two-pass mapping strategy was employed to improve novel splice junction detection. Genes were defined as differentially expressed if they met the criteria of |log2(fold change)| ≥ [X] and Benjamini-Hochberg adjusted P-value < [0.05]. For KEGG pathway enrichment analysis: The hypergeometric test was performed using the clusterProfiler R package, and pathways with an adjusted P-value < 0.05 were considered significantly enriched. These are now clearly described as two independent analytical steps with their respective statistical criteria.

### 2.4 Western blotting assay

Hippocampal tissues were harvested from each mouse and homogenized in 200 μL of PRO-PREP lysis buffer, followed by centrifugation. Protein concentration was quantified using a DC Protein Assay Kit (Bio-Rad) in accordance with the manufacturer’s instructions.10 μg protein samples were boiled for 10 min and separated by SDS–polyacrylamide gel electrophoresis (PAGE) under 80 V for 30 min, followed by 120 V electrophoresis for 30 min. Proteins were then transferred to a polyvinylidene fluoride (PVDF) membrane at 300 mA for 90 min. The membrane was first washed in phosphate-buffered saline (PBS) with Tween 20, followed by blocking in 5% bovine serum albumin (BSA) for 2 hours. The membrane was then incubated in the primary antibody dilution (**Table 2**) at 4 °C overnight. On the next day, the membrane was washed in PBS and was incubated in Secondary antibodies were added for 2 hours. Antibodies were diluted according to the manufacturer’s instructions. The integrated gray values of the target bands were quantified using ImageJ (National Institutes of Health, Bethesda, USA).

**Table 2:** List of antibodies used, source, and dilution information.

Antibody	Company	Lot number	Dilution	Source
NR2A	Proteintech, Wuhan, China	28525-1-AP	1/2000	Rabbit
NR2B	Proteintech, Wuhan, China	21920-1-AP	1/1000	Rabbit
GluA1	Cell signaling, MA, USA	#13185T	1/2000	Rabbit
PSD95	Cell signaling, MA, USA	#2507S	1/1000	Rabbit
IL-1β	Cell signaling, MA, USA	#12242T	1/1000	Rabbit
IL-6	Cell signaling, MA, USA	#12912S	1/1000	Rabbit
IL-17A	Cell signaling, MA, USA	#13838S	1/1000	Rabbit
TNF-α	Cell signaling, MA, USA	#3707S	1/1000	Rabbit
IκB-α	Cell signaling, MA, USA	#4814S	1/1000	Rabbit
IKK-α	Cell signaling, MA, USA	#2682S	1/1000	Rabbit
p65 NF-kB	Cell signaling, MA, USA	#8242S	1/1000	Rabbit
p-IκBα	Cell signaling, MA, USA	#2859S	1/2000	Rabbit
p-IKKα/β	Cell signaling, MA, USA	#2697S	1/1000	Rabbit
p-p65 NF-kB	Cell signaling, MA, USA	#3033S	1/1000	Rabbit
β-actin	Proteintech, Wuhan, China	66009-1-Ig	1/2000	Rabbit
Second-Antibody IgG (H+L)	Servicebio, Wuhan, China	GB23303	1/10000	Goat
Second-Antibody IgG (H+L)	Proteintech, Wuhan, China	RGAR401	1/10000	Rabbit

### 2.5 Golgi-Cox staining

Golgi-Cox staining was performed using a rapid GolgiStain Kit (FD NeuroTech, USA) to characterize potential alterations in neuronal dendritic spine density and morphology. Whole brains were immersed in the staining solution at room temperature, protected from light, for 7 days, in accordance with the manufacturer’s protocol. The tissues were then sectioned at 60 µm. Images were acquired using a confocal microscope (LSM 880, Zeiss, Germany). We have clarified that for the Golgi staining analysis; we used 3 animals per group. From each animal, we randomly selected approximately 6 well-impregnated neurons (totaling 18 neurons per group) for morphological reconstruction. For dendritic spine analysis, we analyzed 18 dendritic segments (e.g., 10 µm length) from the secondary or tertiary dendrites for each selected neuron. Neurons were selected based on the following criteria: (1) complete impregnation of the soma and dendrites; (2) location within the CA1,CA3 and DG; (3) absence of overlapping with other stained cells; and (4) intact primary dendrites. To minimize bias, the experimenter performing the imaging and morphological analysis was blinded to the experimental groups. Brain sections were coded prior to analysis, and the code was broken only after the data collection was completed.

### 2.6 Hippocampal slices preparation and electrophysiological recording

#### 2.6.1 Preparation of brain slices

Nine mice were randomly selected from each group for whole-cell patch-clamp recording. One control group was abandoned due to a slicing error. After respiratory anesthesia with isoflurane, the mice were decapitated, and their brains were quickly immersed in artificial cerebrospinal fluid (ACSF, in mM: 124 NaCl, 2.5 KCl, 1.25 NaH_2_PO_4_·H_2_O, 1.3 MgSO_4_·7H_2_O, 2.6 NaHCO_3_, 10 glucose, 2 CaCl_2_, pH = 7.4, 350 mOsm osmotic pressure) saturated with oxygen mixture (95%O_2_ + 5%CO_2_) at 0–4 °C. The brains were then cut into coronal slices 300 μm thick each using a vibrating slicer (VT1200s, Leica, Germany) and incubated at a constant temperature of 35.5°C for 0.5 h before patch-clamp recording (41–43).

#### 2.6.2 Patch-clamp recording

Patch-clamp recordings were performed on hippocampal brain slices in oxygenated ACSF at room temperature. The CA1 regions of the hippocampus within the slices were visualized using a BX51WI microscope (Olympus, Tokyo, Japan) at high magnification (40×). Before recording sEPSCs/EPSCs, 150 μM picrotoxin (PTX, MedChemExpress, Weehawken, USA) was added to ACSF to inhibit gamma-aminobutyric acid (GABA) receptor-mediated inhibitory synaptic transmission. The recording electrodes were formed using borosilicate glass tubes (O.D: 1.5 mm, I.D: 1.17 mm, Sutter Instruments, CA, USA) using a Flaming-Brown puller (P-1000, Sutter Instruments, CA, USA). The electrode tips were filled with pipette solution (in mM: 0.3Na_2_-GTP, 2EGTA, 10HEPES, 2Mg-ATP, 8NaCl, 120KMeSO_4_, pH = 7.3-7.4, 340 mOsm osmotic pressure), and the electrode resistance was 3–6 MΩ. When recording EPSCs, the concentric bipolar or matrix (100 μm) stimulating electrodes (FHC) were placed in the Schaffer collateral, and the recording electrodes were placed in the CA1; CA1 pyramidal neurons were identified under differential contrast/infrared illumination by their location in the cell body layer and by their pyramidal shape, stimulating intensity of the test pulse (0.2–1.0 mA, duration 0.1 ms). The EPSCs/paird-pulse ratio (PPR, IPI 50 ms) and sEPSCs were recorded using a MultiClamp 700B (Molecular Devices, Foster City, CA, USA) in voltage clamp mode with a holding potential of -70 mV. The low-pass filter was set to 10 kHz, and the series resistance was monitored at 2 min intervals. Recordings with a series resistance change of more than 20% were discarded. Signals were digitized using a Digidata 1550B (Molecular Devices, Foster City, CA, USA) at a sampling frequency of 10 kHz.

#### 2.6.3 LTP recording

Field-evoked excitatory postsynaptic potentials (fEPSPs) were recorded at the stratum radiatum of the CA1 region using a MultiClamp 700B (Molecular Devices, Foster City, CA, USA). For field recording, a glass microelectrode filled with 2 M NaCl was used, and the bath solution routinely contained 100 μM picrotoxin, a γ-aminobutyric acid type A (GABAA) receptor antagonist. Initially, the stimulating intensity of the test pulse (0.2–1.0 mA, duration 0.1 ms) was adjusted. Using gradient increasing electrical stimulation to stimulate Schaffer collateral, a series of equal gradient gradients are set for stimulation intensity, with 30 second intervals between each stimulation intensity. Each intensity is recorded three times, and the average slope of fEPSP is taken to construct the input-output (I/O) curve. The stimulating intensity that produced 30–50% of the maximal fEPSP slope was taken as the optimal stimulating intensity and maintained at the same level in subsequent recordings. Three average responses were obtained to estimate the fEPSP slope. In our experiments, high-frequency stimulation (HFS) was administered after establishing a stable baseline for 15–20 min. HFS consisted of 10 strings, each with five pulses at a frequency of 100 Hz, a wave width of 0.2 ms, and an interval of 200 ms. (44). Recordings and analyses were performed in a blinded manner.

### 2.8 Data analysis

Data for sEPSCs were acquired using Clampfit 10.5 software (Molecular Devices, Foster City, CA, USA). Minianalysis 6.0 software (Synaptosoft Inc., Decatur, GA, USA) was used to extract 2–4 min segments of the recordings for the analysis of the frequency and amplitude of sEPSCs. ImageJ Fiji software was used to analyze the fluorescence intensity levels of proteins and dendritic spine counts (Wayne Rasband, NIH, USA). All data analyses were performed by investigators blinded to the experimental group assignments. The data from the behavioral, WB, and Golgi staining experiments were analyzed on a per-animal basis (n=number of animals), while the data from the patch-clamp recording experiments were analyzed on a per-cell basis (n=number of animals/cells). The data from the LTP experiments were analyzed on a per-brain slice basis (n=number of brain slices). Behavioral data are presented as mean ± SD, and other data are presented as mean ± SEM. All data were analyzed using one-way analysis of variance (ANOVA), followed by Tukey’s multiple comparisons test for three independent groups. Before conducting one-way ANOVA, this study evaluated the homogeneity of variance of each group of data using Levene test. The results showed that there was no statistically significant difference in variance among the groups (p>0.05), and the data met the assumption of homogeneity of variance. Adobe Illustrator CC2017 and GraphPad were used to create and analyze the experimental diagrams.

## 3 Results

### 3.1 Swimming exercise improves the decline in spatial memory caused by LPS

In the present study, the Y-maze spontaneous alternation test was initially performed. As illustrated in **Figures 1B, C**, the mean correct alternation rate of LPS-treated mice was significantly lower than that of the untreated control group (Control: 35.12 ± 9.48%, n=12; LPS: 24.71 ± 9.10%, n=12; p<0.05). However, concurrent administration of LPS and swimming exercise have a trend to ameliorate this deficit (Swim + LPS: 32.89 ± 9.38%, n=12, p =0.096). Furthermore, no significant difference was observed between the Swim+ LPS group and the Control group (p>0.05), indicating that swimming intervention substantially alleviated LPS-induced cognitive impairment.

Subsequently, the novel arm exploration experiment was conducted using a Y maze. As shown in **Figure 1C**, the mean residence time for control mice was 73.31 ± 32.77 s (n=12) in the novel arm, whereas LPS-treated mice exhibited a significant reduction to 41.47 ± 19.65 s (n=12; Control VS LPS: p<0.05). Subsequent swimming intervention significantly improved the residence time in LPS-treated mice (Swim + LPS: 63.07 ± 25.97 s, n=12). Additionally, the residence time difference between the Swim + LPS and Control group was not significant (p>0.05), demonstrating that swimming exercise effectively alleviated the LPS-induced reduction in residence time.

Regarding the Morris Water Maze (MWM) results (**Figures 1D, E**), swimming exercise significantly attenuated the LPS-induced increase in latency to first entry into the target quadrant (Control: 13.86 ± 11.31 s, n=6; LPS: 151.91 ± 63.92 s, n=6; Swim+LPS: 26.30 ± 26.58 s, n=6; Control vs. LPS: p<0.0001; LPS vs. Swim+LPS: p < 0.001; Control vs. Swim+LPS: p>0.05). Swimming have no effects on distance in zone-quadrant SW (Control: 33.15 ± 11.98 m, n=6; LPS: 19.00 ± 6.39 m, n=6; Swim+LPS: 30.12 ± 10.40 m, n=6; Control vs. LPS: p>0.05; LPS vs. Swim+LPS: p>0.05; Control vs. Swim + LPS: p>0.05). Swimming also significantly mitigated the LPS-induced decrease in the total swimming distance (Control: 86.42 ± 29.52 m; LPS: 49.28 ± 11.83 m; Swim+LPS: 82.51 ± 17.7 m; Control vs. LPS: p<0.05; LPS vs. Swim+LPS: p<0.05; Control vs. Swim+LPS: p>0.05). Swimming has no effects on the swimming speed (Control: 0.65 ± 0.05 cm/s; LPS: 0.64 ± 0.12 cm/s; Swim+LPS: 0.61 ± 0.09 cm/s), and time spent in the target quadrat (Control: 134.07 ± 37.93 s; LPS: 124.01 ± 29.77 s; Swim+LPS: 122.79± 3.50 s).

In the Barnes maze test (**Figures 1F, G**), swimming exercise have no effect on the LPS-induced reduction in the number of entries into the target hole (Control: 9.00 ± 7.65, n=5; LPS: 1.00 ± 1.55, n=6; Swim+LPS: 4.80± 3.56, n=5; Control vs. LPS: p<0.05; LPS vs. Swim+LPS: p>0.05; Control vs. Swim+LPS: p>0.05). Furthermore, swimming has not reduced the LPS-induced elevation in latency to first entry into the target hole (Control: 14.83 ± 5.74 s, n=5; LPS: 62.09 ± 38.86 s, n=6; Swim+LPS: 24.46 ± 13.82 s, n=5; Control vs. LPS: p<0.05; LPS vs. Swim+LPS: p>0.05; Control vs. Swim+LPS: p>0.05).

### 3.2 Swimming exercise is associated with alterations in TNF-α/IL-17/NF-κB pathways to attenuate LPS-induced decline in spatial memory

Given that LPS administration impairs spatial memory in mice, whereas swimming exercise mitigates this LPS-induced cognitive decline, we conducted mRNA sequencing (mRNA-seq) analysis on hippocampal tissues from Control, LPS, and Swimming+LPS groups to elucidate the underlying mechanisms. As shown in **Figures 2A, B**, we identified 324 DEGs (245 upregulated and 79 downregulated) between the LPS and Control groups, and 194 DEGs (136 upregulated and 58 downregulated) between the Swim+LPS and LPS groups, using thresholds of adjusted P-value < 0.05 and |log2FC| > 1.Kyoto Encyclopedia of Genes and Genomes (KEGG) enrichment analysis of differentially expressed genes (DEGs) between the LPS and Control groups is presented in **Figure 2C**; corresponding results comparing the Swimming treatment group with the LPS group are shown in **Figure 2D**. Comparative analysis of KEGG pathways between the LPS and Control groups and between the Swimming and LPS groups revealed significant alterations implicating the TNF-α/IL-17/NF-κB signaling pathways.

**Figure 2:**
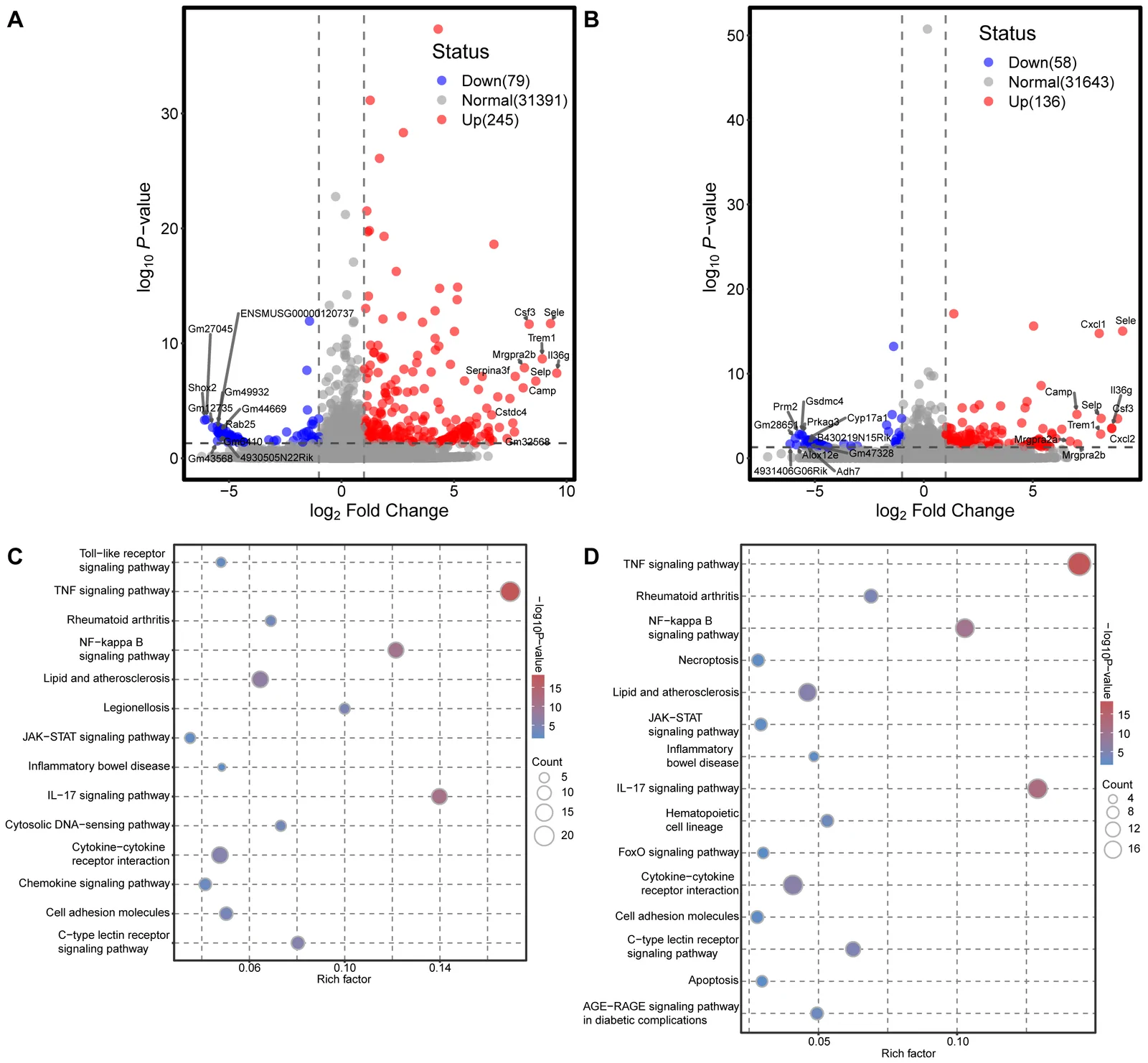
Comparative analysis of signaling pathways in the hippocampus across the three groups. **(A)** Volcano plots between the LPS and control groups in the hippocampus. **(B)** Volcano plots between the Swim + LPS and LPS groups in the hippocampus. **(C)** Bubble plot illustrating the differences in signaling pathways between the LPS and control groups in the hippocampus. **(D)** Bubble chart depicting the comparison of signaling pathways in the hippocampus between the Swim + LPS and LPS groups.

### 3.3 Swimming exercise reduces the levels of IL-17/NF-κB signaling pathway caused by LPS

We used the WB method to detect the relative protein expression levels of TNF-α/IL-17/NF-κB signaling pathways, including IL-6/IL-1β/IL-17a/TNF-α/IκB/IKK and p65 NF-κB in the hippocampus. The experimental results showed that compared with the control group, the relative expression levels of IL-6/IL-17a/TNF-α/p65 NF-κB were significantly higher in the LPS group than in the control groups, and the Swim+LPS group could alleviate this trend, except for TNF-α (IL-6: Control: 1.00 ± 0.07, n=4; LPS: 1.80 ± 0.15, n=4; Control vs LPS p<0.001, Swim+LPS: 1.40 ± 0.05, n=4), LPS VS Swim+LPS p<0.05, **Figure 3A**; IL-17a:Control: 1.00 ± 0.13, n=4; LPS: 1.64 ± 0.09, n=4; Control vs LPS p<0.01, Swim+LPS: 1.24 ± 0.08, n=4; LPS VS Swim+LPS: p<0.05, **Figure 3C**; TNF-α: Control: 1.00 ± 0.04, n=4; LPS: 2.16 ± 0.20, n=4; Control vs LPS p<0.01, Swim+LPS: 1.80 ± 0.25, n=4), LPS VS Swim+LPS: p>0.05, Control VS Swim+LPS, p<0.05, **Figure 3D**; p65:Control: 1.00 ± 0.06, n=4; LPS: 1.28 ± 0.06, n=4; Control vs LPS p<0.05, Swim+LPS: 0.99 ± 0.07, n=4; LPS VS Swim+LPS p<0.05, **Figure 3E**. There was no significant difference in IL-1β, IκB, and IKK levels between the control, LPS, and Swim+LPS groups (n=4 for each group).

**Figure 3:**
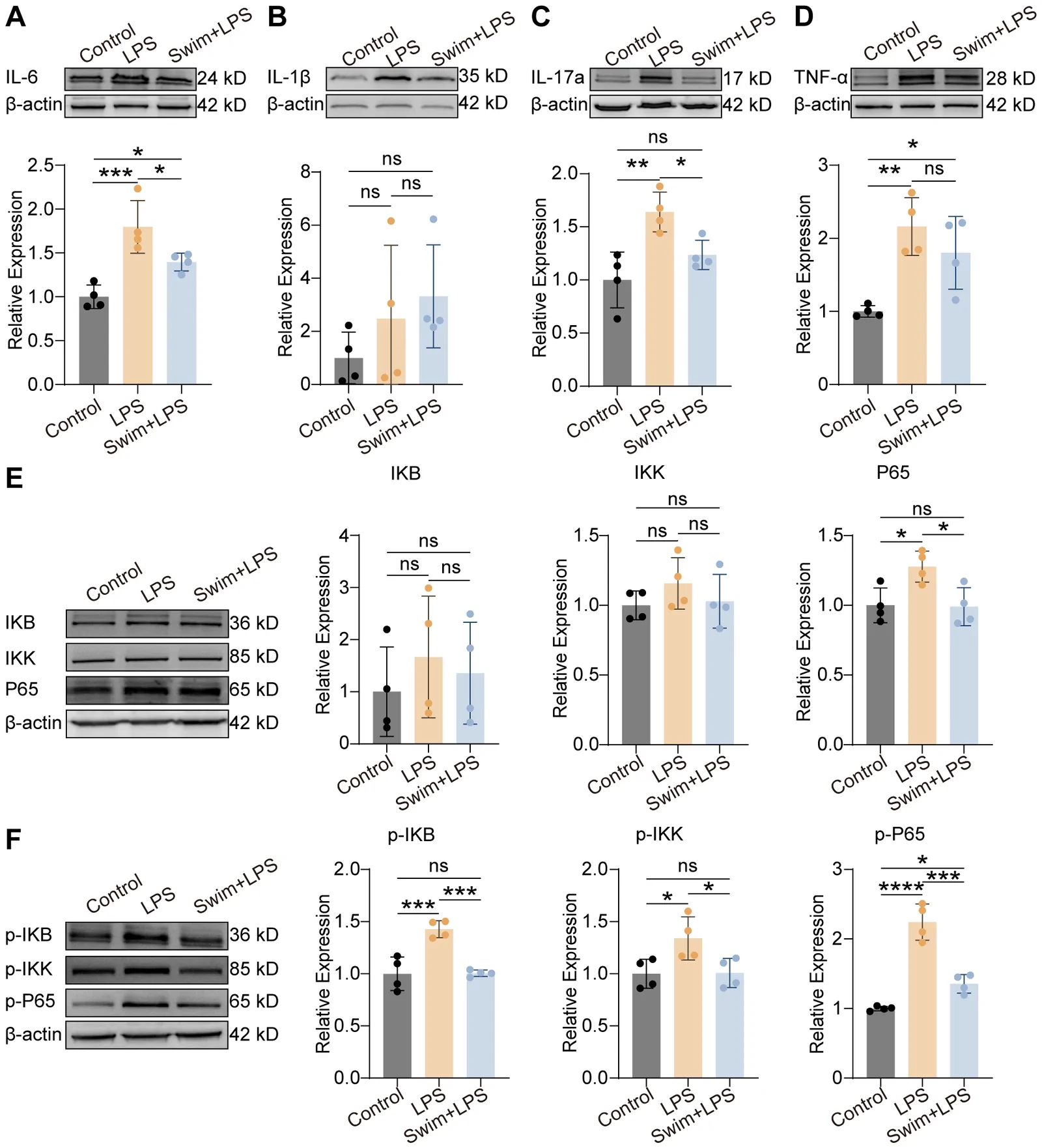
Swimming exercise mitigates the upregulation of inflammatory factor expression and activation of the NF-κB pathway induced by LPS. Representative western blot (WB) band image (top) and corresponding quantification chart (bottom) illustrating that swimming significantly inhibits LPS-induced expression of IL-6 (n=4 for each group) **(A)** and IL-17a (n=4 for each group) **(C)**, but does not alter the expression of IL-1β (n=4 for each group) **(B)**. **(D) **The administration of LPS resulted in an increased expression of TNF-α, whereas swimming did not affect the LPS-induced expression of TNF-α (n=4 for each group). **(E)** Swimming exercise suppresses the NF-κB signaling pathway by inhibiting the activation of NF-κB p65 (right) induced by LPS without affecting IκB and IKK (middle) (n=4 for each group). **(F)** Swimming exercise suppresses the phosphorylated NF-κB-related proteins by inhibiting the activation of p-IκB/p-IKK and p-P65 induced by LPS (n=4 for each group). *p < 0.05.**p < 0.01, ***p < 0.001, ****p < 0.0001.

Given that phosphorylated NF-κB-related proteins (IκB/IKK and p65) serve as direct indicators of the NF-κB signaling pathway’s activation status, we conducted a quantitative analysis of the phosphorylation levels of these three proteins. The findings indicated that swimming markedly counteracted the LPS-induced elevation in phosphorylation levels (p-IκBα: control 1.00 ± 0.08, n=4; LPS 1.43 ± 0.04, n=4; swim+LPS 1.01 ± 0.02, n=4; p-IKKα/β: control 1.00 ± 0.07, n=4; LPS 1.34 ± 0.10, n=4; swim+LPS 1.01 ± 0.07, n=4; p-P65: control 1.00 ± 0.02, n=4; LPS 2.24 ± 0.13, n=4; swim+LPS 1.36 ± 0.07, n=4).

### 3.4 Swimming exercise improves the decrease in the levels of synaptic function-related proteins caused by LPS

Western blotting was used to assess the relative expression levels of GluA1, NR2A, NR2B, and PSD95 in the hippocampus. The results demonstrated that the relative expression level of GluA1 was significantly reduced in the LPS group compared to that in the control group. This decrease was not ameliorated in the Swim+LPS group (GluA1: Control: 1.00 ± 0.22, n=4; LPS: 0.33 ± 0.03, n=4; Swim+LPS: 0.62 ± 0.08, n=4; Control vs. LPS: p<0.05; LPS vs. Swim+LPS: p>0.05; Control vs. Swim+LPS: p>0.05). In contrast, no statistically significant differences were observed in the expression levels of NR2A, NR2B, or PSD95 among the control, LPS, and Swim+LPS groups (**Figure 4A**).

**Figure 4:**
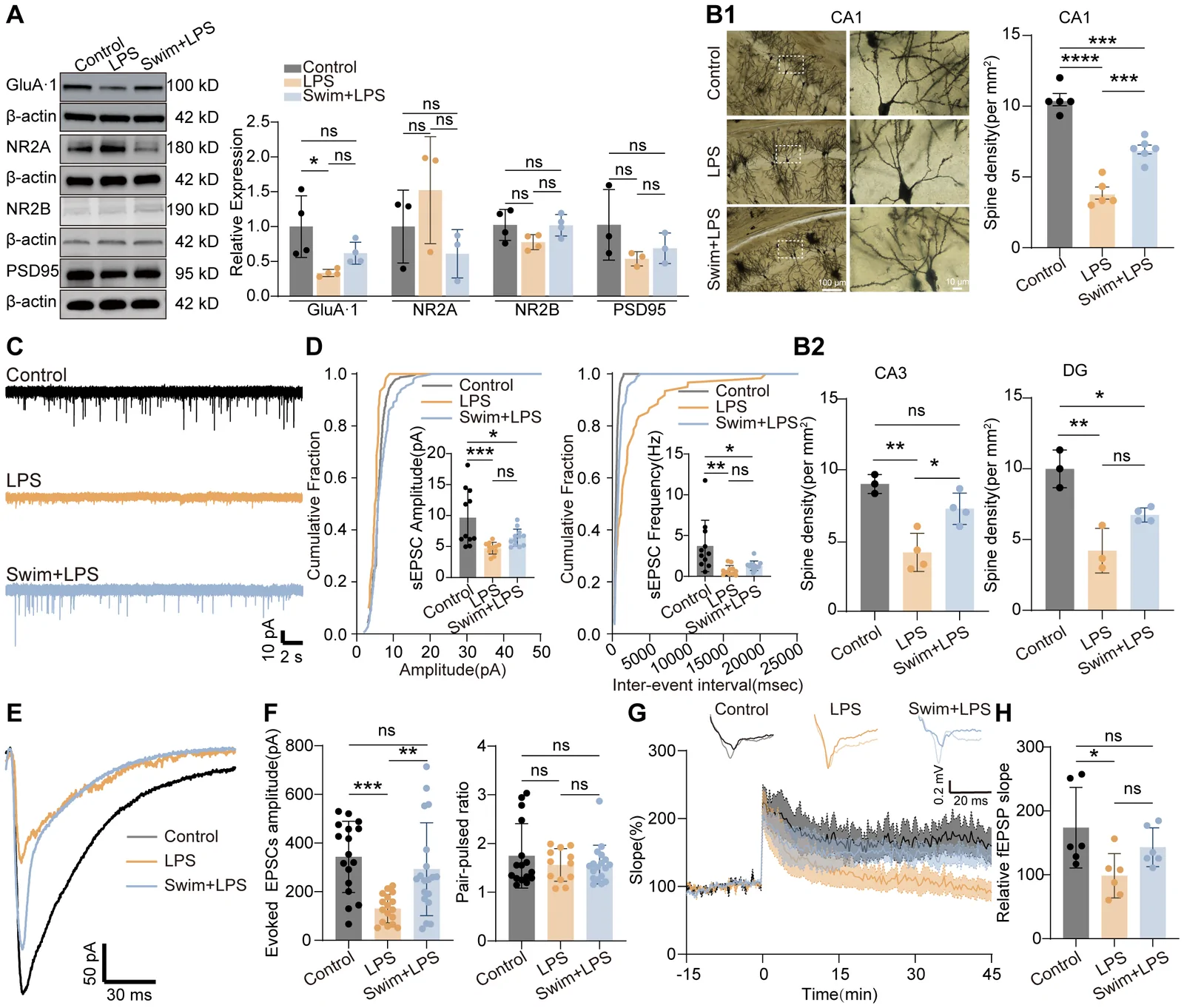
Swimming ameliorates both structural and functional synaptic impairments induced by LPS. **(A)** Western blot analysis illustrating synaptic protein expression in the hippocampal tissues of the three groups of mice (left), accompanied by the corresponding statistical analysis in column charts (right) (n=4 for each group on GluA.1; n=4 for each group on NR2A; n=4 for each group on NR2B; n=4 for each group on PSD95). **(B1)** Golgi staining images of the CA1 region in the hippocampal tissues of the three groups of mice (left), with bar charts depicting dendritic spine density (right, n=5, 5, 6 for each group respectively)**. (B2)** Bar charts depicting dendritic spine density of CA3 (right, n=3, 3, 4 for each group respectively) and DG) (left, n=3, 3, 4 for each group respectively). **(C)** Representative traces of sEPSC recordings from pyramidal neurons in the CA1 region of the hippocampus of the three groups of mice. **(D)** Cumulative distribution curves and bar charts of amplitudes (left, n=3/5/11,3/5/11, 3/5/12 for each group respectively) and inter-event intervals (right, n=3/5/11, 3/5/11, 3/5/12 for each group respectively). **(E)** Representative traces of evoked EPSCs and **(F)** amplitude (left, n=3/4/17, 3/6/17, 3/6/19 for each group respectively) and PPR(right, n=3/6/16, 3/6/12, 3/6/17 for each group respectively) statistical bar charts of hippocampal tissue in the three groups of mice **(G)** Representation of fEPSP traces in the hippocampus of the three groups of mice before and after HFS, with dark-colored lines indicating baseline curves and light-colored lines indicating fEPSP curves 45 min post-HFS (top), along with a line graph illustrating changes in the slope during the LTP induction process (bottom). **(H)** Statistical analysis bar chart of the slope of fEPSP in the hippocampus of the three groups of mice 45 min after HFS (n=6 for each group)., *p < 0.05, **p < 0.01, ***p < 0.001, ****p < 0.0001.

### 3.5 Swimming effects on the LPS induced damage to CA1 excitatory synaptic transmission and LTP

Hippocampal neurons were subjected to Golgi staining to investigate the impact of LPS and Swim+LPS during development on dendritic spine density within the hippocampal CA1 regions. The results revealed that LPS decreased the density of dendritic spines, and swimming alleviated the decrease in dendritic spine density caused by LPS(CA1:Control: 10.47 ± 0.43/10 µm, n=5(n = number of animals); LPS: 4.00 ± 0.55/10 µm, n=5; Control VS LPS: p*<*0.0001;Swim+LPS:6.89 ± 0.32/10 µm;n=5, LPS VS Swim+ LPS: p<0.001). To verify the effect of swimming exercise on LPS-induced hippocampal dysfunction, we used whole-cell patch clamp recording to record sEPSCs in CA1 neurones (**Figures 4C, D**). We found that compared with the control group, the frequency and amplitude of LPS were reduced (Amplitude: Control group: 9.65 ± 1.39 pA, n=10 neurons from 5 slices, n=3; The LPS group had 4.74 ± 0.29 pA, n=11 neurons from 6 slices, n=3; Control VS LPS: *p* < 0.001; Frequency: Control group: 3.72 ± 0.95 Hz, n=10 neurons; The LPS group had 0.74 ± 0.18 Hz, n=11; Control vs LPS: p<0.01), while swimming+LPS can reverse the decrease in amplitude and frequency of sEPSCs caused by LPS (Amplitude: Swim+LPS group: 6.43 ± 0.40 pA, n=10 neurons from 5 slices, n=3; Control VS Swim+LPS: p<0.05; LPS VS Swim+LPS: p>0.05; Frequency: Swim+LPS group: 1.31 ± 0.17 Hz, n=10 neurons from 5 slices, n=3; Control VS Swim+LPS: p<0.05; LPS VS Swim+LPS: p>0.05).

To further verify the effect of swimming exercise on LPS-induced hippocampal dysfunction, we used patch clamp whole cell recording to record the excitatory postsynaptic currents (EPSCs) induced by Schaffer collateral to CA1 (**Figures 4E, F**). We found that compared with the control group, the amplitude of EPSCs decreased at the same stimulation intensity with LPS (Control group: 343.38 ± 35.42 pA, n=17 neuron from 4 slices, n=3); The LPS group had 131.19 ± 14.22 pA, n=17 neurons from 6 slices, n=3); Control VS LPS:p<0.001), swimming combined with LPS can reverse the decrease in EPSCs amplitude caused by LPS (Swim+LPS: 292.85 ± 43.80 pA, n=19 neurons from 6 slices, n=3; Control VS Swim+LPS: p>0.05;LPS VS Swim+LPS: p<0.01)。

Initially, fEPSPs were monitored in the CA1 region of hippocampal slices from wild-type control mice by electrically stimulating the Schaffer collateral commissural pathway. HFS enhanced fEPSP slopes to 150–230% of basal levels in wild-type control mice, being evident at 40 min after HFS; in other words, HFS induced Schaffer collateral/CA1 LTP in control mice (**Figure 4G**). HFS-induced potentiation of fEPSP slopes in LPS mice was significantly lower than that in control mice (Control: 173.82 ± 25.77 (n= 6 slices, n=3); LPS: 98.56 ± 14.13 (n= 6 slices, n=3); Control VS LPS: p<0.05, **Figure 4H**). This implies that Schaffer collateral/CA1LTP is suppressed by LPS administration. Although the suppression of long-term potentiation (LTP) in LPS-treated mice was not neutralized by swimming exercise (Swim+LPS: 143.22 ± 12.37 (n= 6 slices, n=3); LPS vs. Swim+LPS: p > 0.05; **Figure 4H**), no significant difference was observed between the control group and the Swim+LPS group (p > 0.05, **Figure 4H**).

## 4 Discussion

This study utilized juvenile male C57BL/6 mice to establish a model of cognitive impairment induced by lipopolysaccharide (LPS). Spatial memory performance was evaluated, and a swimming exercise intervention was conducted to investigate its effects on spatial memory and the expression of inflammatory and synaptic proteins within the hippocampus, a critical brain region for spatial memory. The main findings are as follows: (1) LPS administration significantly impaired spatial memory in juvenile mice, whereas the swimming exercise intervention alleviated this LPS-induced deficit. (2) In comparison to control mice, LPS-treated mice exhibited significantly elevated hippocampal expression levels of key proteins in the NF-κB pathway (NF-κB p65, p-IκBα, p-IKKα/β, and p-P65) and pro-inflammatory cytokines (IL-6, IL-17). The swimming exercise significantly reduced the expression of these inflammatory factors within the hippocampus. (3) The hippocampal expression levels of GluA1 were markedly reduced in LPS-treated mice compared to controls. The swimming exercise normalized the expression of these synaptic proteins. (4) LPS significantly decreased the frequency and amplitude of spontaneous excitatory postsynaptic currents (sEPSCs) in the CA1 region, as well as evoked EPSCs and long-term potentiation (LTP) in the Schaffer collateral-CA1 pathway. The swimming intervention mitigated these electrophysiological impairments.

The forced swimming can act as a stressor (45). However, our protocol involved a chronic swimming regimen (60 min/day for 4 weeks). Chronic swimming protocols typically induce physiological adaptations similar to voluntary exercise, such as reduced basal corticosterone levels and improved cardiovascular function, distinguishing them from acute stress responses (46, 47). The behavioral findings of this study corroborated that swimming exercise markedly enhanced the spatial memory capabilities of LPS-treated developing mice. This outcome aligns with existing evidence indicating that aerobic exercise can improve cognitive function. (5, 6). The research focused on developing mice, a stage characterized by the vigorous structural and functional maturation of the hippocampus, the primary brain region responsible for spatial memory. Inflammation induced by LPS during this critical period may result in more severe and enduring damage to cognitive development. (48). Recent studies have shown that intraperitoneal administration of LPS at doses ranging from 0.1 to 0.5 mg/kg results in deficits in spatial learning and memory, as well as changes in neuroprotective biomarkers linked to memory function. (7, 15, 16). The observation that swimming attenuates such impairments not only verifies the anti-inflammatory and cognitive-protective properties of exercise, but also highlights the potential value of exercise-based strategies for maintaining cognitive development in juveniles exposed to inflammatory stress.

RNA sequencing analysis demonstrated that the gene expression profiles in the hippocampus of the control, LPS, and swim + LPS groups exhibited a strong correlation with inflammatory responses, specifically involving the TNF-α, NF-κB, and IL-17 pathways. This provides a transcriptomic foundation for further investigation into the molecular mechanisms. Western blot analysis corroborated these findings, showing that LPS treatment significantly increased the expression of pro-inflammatory factors (IL-6, IL-17, TNF-α) and key molecules within the NF-κB pathway (NF-κB p65) in the hippocampus. The NF-κB pathway serves as a central regulator of the inflammatory response; its abnormal activation can lead to the extensive release of inflammatory factors, which subsequently damage hippocampal neurons and synaptic function, ultimately resulting in cognitive impairment. (49, 50). LPS was observed to increase the expression levels of p-IκBα and p-IKKα/β relative to the blank group, while it did not alter the protein levels of IκB and IKK. This suggests that LPS influences only the post-translational modifications of IκB and IKK. Conversely, swimming exercise significantly downregulated the expression of these inflammatory factors, with the exception of TNFα-related molecules. This indicates that the consistent with the hypothesis that swimming exercise may exert anti-inflammatory effects through modulation of the NF-κB pathway, although direct causal evidence remains to be established. These findings are consistent with previous research demonstrating that aerobic exercise can suppress the activation of the NF-κB pathway in the central nervous system, thereby mitigating neuroinflammation. (51, 52). The present study delineates the regulatory effect within the context of LPS-induced inflammation in developing mice, thereby enhancing the understanding of exercise-induced anti-neuroinflammation. Furthermore, recent evidence has strengthened the understanding of the complex regulatory network of neuroinflammation in cognitive dysfunction. Chemokines are identified as crucial mediators in chronic neuroinflammation, closely associated with inflammatory signaling and neuronal dysfunction. (53); The inhibition of ferroptosis in hippocampal neurons can effectively maintain cognitive function under conditions of neurotoxic stress. (54); The targeting of CD44 markedly reduces neuroinflammation and safeguards dopaminergic neurons, highlighting the interaction between immune and membrane components in central inflammatory responses. (55); Forsythoside B mitigates neuroinflammation and neuronal apoptosis through the inhibition of NF-κB and p38-MAPK pathways, concurrently activating Nrf2 signaling. (56); Microglial metabolic reprogramming, characterized by citrate accumulation, facilitates pro-inflammatory polarization following brain injury, thereby unveiling a novel metabolic aspect of neuroinflammation. (57). These findings collectively broaden the mechanistic understanding of neuroinflammation and suggest that the cognitive benefits of swimming may involve pathways including IL-17/NF-κB signaling, warranting further investigation to establish causal relationships.

Systemic LPS injection triggers microglial IL-6 and acute cognitive dysfunction in APP/PS1 mice (58). Elevated IL-6 levels are associated with decreased spatial memory in rodents (59–61). This study found that LPS increases IL-6 levels in the hippocampus, whereas swimming exercise reduces the LPS-induced increase in IL-6 levels in the hippocampus. This indicates that swimming exercise can improve spatial memory involving regulation of neuroinflammation, although the specific pathways remain to be formally demonstrated. The plasticity of glutamatergic synapses was reduced in the absence of IL-17, resulting in impaired long-term potentiation in the hippocampus. Conversely, IL-17 enhanced the production of brain-derived neurotrophic factor by glial cells, and the exogenous provision of this factor rescued the synaptic and behavioral phenotypes of IL-17-deficient animals (14).

Synaptic function and plasticity are the cellular and molecular bases of learning and memory. The present study found that LPS treatment significantly downregulated the expression of synaptic function-related proteins, GluA1, in the hippocampus; GluA1-AMPARs are the key receptors involved in excitatory synaptic transmission, and their expression levels directly affect the efficiency of synaptic transmission. Consistent with this, electrophysiological results showed that LPS treatment decreased the frequency and amplitude of spontaneous excitatory postsynaptic currents (sEPSCs) in the CA1 region, as well as the amplitude of evoked EPSCs and LTP in the Schaffer collateral-CA1 pathway. LTP is a classic cellular model of learning and memory, and the impairment of LTP directly reflects damage to synaptic plasticity and memory formation ability (62). It should be noted that the LTP recording was conducted for only 45 minutes, which is sufficient to capture early LTP (E-LTP) but insufficient to confirm the effect of swimming on late LTP (L-LTP). Given that L-LTP is closely associated with the long-term maintenance of spatial memory, its investigation will be addressed in our future work.

Importantly, swimming exercise ameliorated the above-mentioned LPS-induced impairments in synaptic function. It upregulated the expression of AMPARs and increased the frequency and amplitude of sEPSCs in the CA1 region, as well as the amplitude of EPSCs and LTP in the Schaffer collateral-CA1 pathway. These results indicate that swimming exercise may mitigate neuroinflammation and directly enhance hippocampal synaptic function and plasticity, which could contribute to its protection of spatial memory. Furthermore, combined with the results of the NF-κB pathway, it can be plausible that swimming exercise may first inhibit the NF-κB pathway to reduce the release of inflammatory factors, thereby reducing the inflammatory damage to synaptic structure and function; at the same time, it may directly regulate the expression of synaptic function-related proteins to enhance synaptic transmission and plasticity. These two pathways work synergistically to achieve a protective effect on spatial memory. However, the specific upstream signaling molecules that mediate the regulation of the NF-κB pathway and synaptic proteins by swimming exercise remain to be further explored. LPS induces changes in the IL-17/NF-kB pathway in mice, potentially affecting chemokine alterations and leading to a decline in spatial memory levels (53).

The present study has important theoretical and practical significance. Theoretically, this study fills the gap in the research on the effect of aerobic exercise on spatial memory under inflammatory conditions in developing individuals, provides observational evidence linking the IL-17/NF-κB pathway and synaptic function in this process, and enriches the molecular mechanisms of exercise-regulated cognitive function. Practically, swimming is a safe and easy-to-implement aerobic exercise. Swimming is a safe and practicable aerobic exercise. The finding that swimming alleviates LPS-induced spatial memory impairment in developing mice suggests a potential non-pharmacological strategy to sustain cognitive development under inflammatory stress in the adolescent period.

This study had certain limitations. First, while our transcriptomic and western blot data consistently implicated the IL-17/NF-κB signaling pathway as a key target of swimming intervention, we did not employ direct pharmacological or genetic manipulation (e.g., IL-17 neutralizing antibodies, NF-κB inhibitors such as BAY 11-7082, or IL-17RA knockout mice) to establish causality. Therefore, whether swimming exerts its cognitive-protective effects primarily through direct modulation of this pathway remains to be formally established. Furthermore, the potential role of swimming-induced systemic factors, such as myokines, BDNF, and PGC-1α, in mediating these effects on the brain should also be tested in our future work. Second, regarding electrophysiological recordings, multiple neurons were sampled from individual animals, and the unit of analysis was the neuron rather than the animal. This approach carries the potential risk of pseudoreplication, as neurons from the same animal may not be fully independent observations. Although we reported the number of animals (n) alongside the number of recorded neurons/slices, future studies should employ mixed-effects models that account for the hierarchical structure of the data (neurons nested within animals) or use animal-level averaged values as the statistical unit to rigorously address this concern. Third, the RNA-seq analysis was performed with three biological replicates per group. While this is a common practice in transcriptomic studies, the relatively small sample size may limit the statistical power to detect differentially expressed genes with modest fold-changes and could increase the likelihood of false-positive findings. To mitigate this concern, we applied stringent filtering criteria (|log2FC| ≥ 1 and FDR < 0.05) and focused on pathways with strong biological prior evidence. Nevertheless, validation of key differentially expressed genes by quantitative real-time PCR (qRT-PCR) in an independent cohort, as well as larger-scale transcriptomic studies, would strengthen the robustness of our transcriptomic conclusions. Fourth, the present study did not explore the effects of different swimming parameters (such as intensity, duration, and frequency) on the outcomes; thus, it was impossible to determine the optimal intervention scheme. Fifth, the absence of a swimming-only control group represents a limitation of this study. Subsequent research should incorporate this group to systematically elucidate the independent baseline effects of swimming and differentiate them from the protective effects against LPS. Additionally, the evidence concerning the inhibition of the IL-17/NF-κB pathway was incomplete and will be investigated in future studies. Sixth, the experimental subjects were restricted to male mice. Fluctuations in endogenous sex hormones in female mice are known to modulate hippocampal plasticity, cognitive performance, and central inflammatory signaling pathways, which may lead to divergent outcomes of swimming exercise intervention between the two sexes. Hence, the present findings cannot be generalized to female mice, and systematic investigations involving female animals are needed in follow-up studies. Lastly, our behavioral assessment and electrophysiological recordings were confined to the immediate post-intervention period. The LTP recording was conducted for only 45 minutes, which is sufficient to capture early LTP (E-LTP) but insufficient to confirm the effect of swimming on late LTP (L-LTP), a process that requires *de novo* protein synthesis and is associated with long-term memory consolidation. Whether the cognitive benefits of swimming persist over extended timeframes (e.g., weeks to months after intervention cessation) remains to be determined. Longitudinal follow-up studies incorporating delayed behavioral testing and sustained electrophysiological recordings will be valuable to evaluate the durability of exercise-induced neuroprotection. This study exclusively utilized 4-week-old juvenile mice. As brain development status, neuronal activity, and inflammatory microenvironment vary greatly across different life stages, whether the positive effects of swimming exercise can be replicated in adult mice remains to be elucidated. We suggest that future research should further dissect the age-dependent mechanism of swimming-mediated neural regulation and distinguish the functional differences of exercise intervention in developing and mature brain tissues.

This study had certain limitations. First, there is currently a lack of pharmacological evidence to demonstrate that swimming exercise alleviates LPS-induced cognitive deficits by inhibiting the IL-17/NF-κB pathway. Furthermore, the potential role of swimming-induced systemic factors, such as myokines, BDNF, and PGC-1α, in mediating these effects on the brain should also be tested in our future work. Second, the present study did not explore the effects of different swimming parameters (such as intensity, duration, and frequency) on the results; thus, it was impossible to determine the optimal intervention scheme. Third, the absence of a swimming-only control group represents a limitation of this study. Subsequent research should incorporate this group to systematically elucidate the independent baseline effects of swimming and differentiate them from the protective effects against LPS. Additionally, the evidence concerning the inhibition of the IL-17/NF-κB pathway was incomplete and will be investigated in future studies. Fourth, the experimental subjects were restricted to male mice. Fluctuations in endogenous sex hormones in female mice are known to modulate hippocampal plasticity, cognitive performance, and central inflammatory signaling pathways, which may lead to divergent outcomes of swimming exercise intervention between the two sexes. Hence, the present findings cannot be generalized to female mice, and systematic investigations involving female animals are needed in follow-up studies. This study exclusively utilized 4-week-old juvenile mice. Lastly, as brain development status, neuronal activity, and inflammatory microenvironment vary greatly across different life stages, whether the positive effects of swimming exercise can be replicated in adult mice remains to be elucidated. We suggest that future research should further dissect the age-dependent mechanism of swimming-mediated neural regulation and distinguish the functional differences of exercise intervention in developing and mature brain tissues.

In conclusion, the present study demonstrated that swimming exercise alleviated LPS-induced spatial memory impairment in developing mice. These effects were associated with modulation of the hippocampal IL-17/NF-κB pathway and enhancement of synaptic function-related protein expression. While these findings suggest a potential role for the IL-17/NF-κB pathway in the cognitive benefits of swimming, causal relationships remain to be formally established. Future studies can explore the protective effect of swimming exercise under chronic inflammatory conditions, optimize the exercise intervention scheme, and employ pharmacological or genetic approaches to delineate whether swimming influences the IL-17/NF-κB pathway to provide a more solid theoretical basis for the clinical application of exercise intervention in protecting juvenile cognitive development.

## Data Availability

The original contributions presented in the study are publicly available. This data can be found here: DOI is https://doi.org/10.57760/sciencedb.45597; CSTR is https://cstr.cn/31253.11.sciencedb.45597.

## References

[1] Glikmann-Johnston Y Fink KD Deng P Torrest A Stout JC . Spatial memory in Huntington's disease: a comparative review of human and animal data. Neurosci Biobehav Rev. (2019) 98:194–207. doi: 10.1016/j.neubiorev.2019.01.015 30658070

[2] Leuner B Gould E . Structural plasticity and hippocampal function. Annu Rev Psychol. (2010) 61:111–40. doi: 10.1146/annurev.psych.093008.100359 19575621 PMC3012424

[3] Meléndez DM Nordquist RE Vanderschuren LJMJ van der Staay F-J . Spatial memory deficits after vincristine-induced lesions to the dorsal hippocampus. PLoS One. (2020) 15:e0231941. doi: 10.1371/journal.pone.0231941 PMC7173870 32315349

[4] Rappeneau V Castillo Díaz F . Convergence of oxytocin and dopamine signalling in neuronal circuits: insights into the neurobiology of social interactions across species. Neurosci Biobehav Rev. (2024) 161:105675. doi: 10.1016/j.neubiorev.2024.105675 38608828

[5] Wan C Shi L Lai Y Wu Z Zou M Liu Z . Long-term voluntary running improves cognitive ability in developing mice by modulating the cholinergic system, antioxidant ability, and BDNF/PI3K/Akt/CREB pathway. Neurosci Lett. (2024) 836:137872. doi: 10.1016/j.neulet.2024.137872 38889879

[6] Wan C Song X Zhang Z Hu W Chen Y Sun W . Voluntary exercise during puberty promotes spatial memory and hippocampal DG/CA3 synaptic transmission in mice. Cereb Cortex. (2024) 34(1):bhad497. doi: 10.1093/cercor/bhad497 38124544

[7] Choi J-W Jo S-W Kim D-E Paik I-Y Balakrishnan R . Aerobic exercise attenuates LPS-induced cognitive dysfunction by reducing oxidative stress, glial activation, and neuroinflammation. Redox Biol. (2024) 71:103101. doi: 10.1016/j.redox.2024.103101 38408409 PMC10904279

[8] Pulido-Salgado M Vidal-Taboada JM Barriga G-D Solà C Saura J . RNA-Seq transcriptomic profiling of primary murine microglia treated with LPS or LPS + IFNγ. Sci Rep. (2018) 8:16096. doi: 10.1038/s41598-018-34412-9 30382133 PMC6208373

[9] Snow WM Stoesz BM Kelly DM Albensi BC . Roles for NF-κB and gene targets of NF-κB in synaptic plasticity, memory, and navigation. Mol Neurobiol. (2013) 49:757–70. doi: 10.1007/s12035-013-8555-y 24122352

[10] Muhammad T Ikram M Ullah R Rehman SU Kim MO . Hesperetin, a citrus flavonoid, attenuates LPS-induced neuroinflammation, apoptosis and memory impairments by modulating TLR4/NF-κB signaling. Nutrients. (2019) 11(3):648. doi: 10.3390/nu11030648 30884890 PMC6471991

[11] Zhao W Xu Z Cao J Fu Q Wu Y Zhang X . Elamipretide (SS-31) improves mitochondrial dysfunction, synaptic and memory impairment induced by lipopolysaccharide in mice. J Neuroinflamm. (2019) 16:230. doi: 10.1186/s12974-019-1627-9 31747905 PMC6865061

[12] Chen P Guo Z Lei J Wang Y . Pomegranate polyphenol punicalin ameliorates lipopolysaccharide-induced memory impairment, behavioral disorders, oxidative stress, and neuroinflammation via inhibition of TLR4-NF-кB pathway. Phytother Res. (2024) 38:3489–508. doi: 10.1002/ptr.8219 38695373

[13] Di Filippo M Mancini A Bellingacci L Gaetani L Mazzocchetti P Zelante T . Interleukin-17 affects synaptic plasticity and cognition in an experimental model of multiple sclerosis. Cell Rep. (2021) 37:110094. doi: 10.1016/j.celrep.2021.110094 34879272

[14] Ribeiro M Brigas HC Temido-Ferreira M Pousinha PA Regen T Santa C . Meningeal γδ T cell-derived IL-17 controls synaptic plasticity and short-term memory. Sci Immunol. (2019) 4(40):eaay5199. doi: 10.1126/sciimmunol.aay5199 PMC6894940 31604844

[15] Moosavi Sohroforouzani A Shakerian S Ghanbarzadeh M Alaei H . Treadmill exercise improves LPS-induced memory impairments via endocannabinoid receptors and cyclooxygenase enzymes. Behav Brain Res. (2020) 380:112440. doi: 10.1016/j.bbr.2019.112440 31863846

[16] Sohroforouzani AM Shakerian S Ghanbarzadeh M Alaei H . Effect of forced treadmill exercise on stimulation of BDNF expression, depression symptoms, tactile memory and working memory in LPS-treated rats. Behav Brain Res. (2022) 418:113645. doi: 10.1016/j.bbr.2021.113645 34743949

[17] Meeusen R . Exercise and the brain: insight in new therapeutic modalities. Ann Transplant. (2005) 10:49–51. 17037089

[18] Cotman CW Berchtold NC . Physical activity and the maintenance of cognition: learning from animal models. Alzheimers Dement. (2007) 3:S30–7. doi: 10.1016/j.jalz.2007.01.013 19595972

[19] Zhang M Jia J Yang Y Zhang L Wang X . Effects of exercise interventions on cognitive functions in healthy populations: A systematic review and meta-analysis. Ageing Res Rev. (2023) 92:102116. doi: 10.1016/j.arr.2023.102116 37924980

[20] Sanders LMJ Hortobágyi T Karssemeijer EGA Van der Zee EA Scherder EJA van Heuvelen MJG . Effects of low- and high-intensity physical exercise on physical and cognitive function in older persons with dementia: a randomized controlled trial. Alzheimers Res Ther. (2020) 12:28. doi: 10.1186/s13195-020-00597-3 32192537 PMC7082953

[21] Shekarchian M Peeri M Azarbayjani MA . Physical activity in a swimming pool attenuates memory impairment by reducing glutamate and inflammatory cytokines and increasing BDNF in the brain of mice with type 2 diabetes. Brain Res Bull. (2023) 201:110725. doi: 10.1016/j.brainresbull.2023.110725 37543294

[22] Seo DY Lee SR Kim N Ko KS Rhee BD Han J . Humanized animal exercise model for clinical implication. Pflugers Arch. (2014) 466:1673–87. doi: 10.1007/s00424-014-1496-0 24647666

[23] Iemitsu M Miyauchi T Maeda S Tanabe T Takanashi M Matsuda M . Exercise training improves cardiac function-related gene levels through thyroid hormone receptor signaling in aged rats. Am J Physiol Heart Circ Physiol. (2004) 286:H1696–705. doi: 10.1152/ajpheart.00761.2003 14704232

[24] Lin J Shao X Shi T Wang H Zhang P Zhou Y . Exercise alleviates cognitive dysfunction in Alzheimer's disease mice via skeletal muscle-derived extracellular vesicles that enhance plaque clearance by microglia. Nat Aging. (2026) 6:579–96. doi: 10.1038/s43587-026-01075-5 41772068

[25] Elgizawy EI Abo-Elsoud RAA Shaban AM Kamel HFM Al-Amodi HS Abdelsattar S . Comparative neuroprotective and exercise capacity effects of prophylactic intermittent fasting and probiotics in sleep-deprived rats: insights into anti-inflammatory marker modulation and CLOCK gene regulation. Front Pharmacol. (2026) 17:1796727. doi: 10.3389/fphar.2026.1796727 42358374 PMC13290755

[26] Reed MD Yim YS Wimmer RD Kim H Ryu C Welch GM . IL-17a promotes sociability in mouse models of neurodevelopmental disorders. Nature. (2019) 577:249–63. doi: 10.1038/s41586-019-1843-6 31853066 PMC8112727

[27] Oriá RB Murray-Kolb LE Scharf RJ Pendergast LL Lang DR Kolling GL . Early-life enteric infections: relation between chronic systemic inflammation and poor cognition in children. Nutr Rev. (2016) 74:374–86. doi: 10.1093/nutrit/nuw008 PMC4892302 27142301

[28] Kokosi T Flouri E Midouhas E . The role of inflammation in the association between poverty and working memory in childhood. Psychoneuroendocrinology. (2020) 123:105040. doi: 10.1016/j.psyneuen.2020.105040 33197720

[29] Chamaa F Darwish B Nahas Z Al-Chaer ED Saadé NE Abou-Kheir W . Long-term stimulation of the anteromedial thalamus increases hippocampal neurogenesis and spatial reference memory in adult rats. Behav Brain Res. (2021) 402:113114. doi: 10.1016/j.bbr.2021.113114 33417991

[30] Xu Y Liu Y Chen X Xu Q Liu L Liu H . OPTN attenuates the neurotoxicity of abnormal Tau protein by restoring autophagy. Transl Psychiatry. (2022) 12:230. doi: 10.1038/s41398-022-02004-x 35662233 PMC9167278

[31] Talley CE Kahn S Alexander LJ Gold PE . Epinephrine fails to enhance performance of food-deprived rats on a delayed spontaneous alternation task. Neurobiol Learn Mem. (2000) 73:79–86. doi: 10.1006/nlme.1999.3920 10686125

[32] Stefani MR Gold PE . Intrahippocampal infusions of k-atp channel modulators influence spontaneous alternation performance: relationships to acetylcholine release in the hippocampus. J Neurosci Off J Soc For Neurosci. (2001) 21:609–14. doi: 10.1523/jneurosci.21-02-00609.2001 11160440 PMC6763830

[33] Burgess A Dubey S Yeung S Hough O Eterman N Aubert I . Alzheimer disease in a mouse model: MR imaging-guided focused ultrasound targeted to the hippocampus opens the blood-brain barrier and improves pathologic abnormalities and behavior. Radiology. (2014) 273:736–45. doi: 10.1148/radiol.14140245 25222068 PMC4314115

[34] Kraeuter AK Guest PC Sarnyai Z . The Y-maze for assessment of spatial working and reference memory in mice. Methods Mol Biol. (2019) 1916:105–11. 10.1007/978-1-4939-8994-2_10 30535688

[35] Lalonde R . The neurobiological basis of spontaneous alternation. Neurosci Biobehav Rev. (2002) 26:91–104. doi: 10.1016/s0149-7634(01)00041-0 11835987

[36] Lin H Zhang J Dai Y Liu H He X Chen L . Neurogranin as an important regulator in swimming training to improve the spatial memory dysfunction of mice with chronic cerebral hypoperfusion. J Sport Health Sci. (2023) 12:116–29. doi: 10.1016/j.jshs.2022.01.008 35066217 PMC9923430

[37] Barnes CA . Memory deficits associated with senescence: a neurophysiological and behavioral study in the rat. J Comp Physiol Psychol. (1979) 93(1):74–104. doi: 10.1037/h0077579 221551

[38] Chen S Zhou Y Chen Y Gu J . fastp: an ultra-fast all-in-one FASTQ preprocessor. Bioinf (Oxford England). (2018) 34:i884–90. doi: 10.1093/bioinformatics/bty560 30423086 PMC6129281

[39] Dobin A Davis CA Schlesinger F Drenkow J Zaleski C Jha S . STAR: ultrafast universal RNA-seq aligner. Bioinf (Oxford England). (2013) 29:15–21. doi: 10.1093/bioinformatics/bts635 PMC3530905 23104886

[40] Pertea M Pertea GM Antonescu CM Chang TC Mendell JT Salzberg SL . StringTie enables improved reconstruction of a transcriptome from RNA-seq reads. Nat Biotechnol. (2015) 33:290–5. doi: 10.1038/nbt.3122 25690850 PMC4643835

[41] Meng W Wang SH Li DF . Carbachol-induced reduction in the activity of adult male zebra finch RA projection neurons. Neural Plast. (2016) 2016:7246827. doi: 10.1155/2016/7246827 26904300 PMC4745321

[42] Noh K Lee H Choi TY Joo Y Kim SJ Kim H . Negr1 controls adult hippocampal neurogenesis and affective behaviors. Mol Psychiatry. (2019) 24:1189–205. doi: 10.1016/j.ibror.2019.07.753 30651602

[43] Zhang Y Wu J Yan Y Gu Y Ma Y Wang M . SAPAP3 regulates epileptic seizures involving GluN2A in post-synaptic densities. Cell Death Dis. (2022) 13:437. doi: 10.1038/s41419-022-04876-9 35513389 PMC9072407

[44] Kim J-H Michiko N Choi I-S Kim Y Jeong J-Y Lee M-G . Aberrant activation of hippocampal astrocytes causes neuroinflammation and cognitive decline in mice. PLoS Biol. (2024) 22:e3002687. doi: 10.1371/journal.pbio.3002687 38991663 PMC11239238

[45] Molendijk ML de Kloet ER . Coping with the forced swim stressor: current state-of-the-art. Behav Brain Res. (2019) 364:1–10. doi: 10.1016/j.bbr.2019.02.005 30738104

[46] Cox RH Hubbard JW Lawler JE Sanders BJ Mitchell VP . Exercise training attenuates stress-induced hypertension in the rat. Hypertension. (1985) 7:747–51. doi: 10.1161/01.hyp.7.5.747 4030045

[47] Lütgemeier I Luft FC Unger T Ganten U Lang RE Gless KH . Blood pressure, electrolyte and adrenal responses in swim-trained hypertensive rats. J Hypertens. (1987) 5:241–7. doi: 10.1097/00004872-198704000-00017 3611773

[48] Kim J Lee H-J Park J-H Cha B-Y Hoe H-S . Nilotinib modulates LPS-induced cognitive impairment and neuroinflammatory responses by regulating P38/STAT3 signaling. J Neuroinflamm. (2022) 19:187. doi: 10.1186/s12974-022-02549-0 35841100 PMC9288088

[49] Fan X Zhang Y Song Y Zhao Y Xu Y Guo F . Compound Danshen Dripping Pills moderate intestinal flora and the TLR4/MyD88/NF-κB signaling pathway in alleviating cognitive dysfunction in type 2 diabetic KK-Ay mice. Phytomedicine. (2023) 111:154656. doi: 10.1016/j.phymed.2023.154656 36682300

[50] Wei X Zhang F Cheng D Wang Z Xing N Yuan J . Free heme induces neuroinflammation and cognitive impairment by microglial activation via the TLR4/MyD88/NF-κB signaling pathway. Cell Commun Signal. (2024) 22:16. doi: 10.1186/s12964-023-01387-8 38183122 PMC10768134

[51] Dehqanizadeh B Mohammadi ZF Kalani AHT Mirghani SJ . Effect of early exercise on inflammatory parameters and apoptosis in CA1 area of the hippocampus following cerebral ischemia-reperfusion in rats. Brain Res Bull. (2022) 182:102–10. doi: 10.1016/j.brainresbull.2022.02.011 35189318

[52] Liu Y Meng X Tang C Zheng L Tao K Guo W . Aerobic exercise modulates RIPK1-mediated MAP3K5/JNK and NF-κB pathways to suppress microglia activation and neuroinflammation in the hippocampus of D-gal-induced accelerated aging mice. Physiol Behav. (2024) 286:114676. doi: 10.1016/j.physbeh.2024.114676 39181380

[53] Jiang BC Liu T Gao YJ . Chemokines in chronic pain: cellular and molecular mechanisms and therapeutic potential. Pharmacol Ther. (2020) 212:107581. doi: 10.1016/j.pharmthera.2020.107581 32450191

[54] Wang X Chen H Shao N Zhang X Huang C Li X . Protective effect of Aloe-emodin on cognitive function in copper-loaded rats based on the inhibition of hippocampal neuron ferroptosis. Curr Neurovasc Res. (2025) 21:458–71. doi: 10.2174/0115672026348862241003042336 39400027

[55] Wang Y Li L Wu Y Zhang S Ju Q Yang Y . CD44 deficiency represses neuroinflammation and rescues dopaminergic neurons in a mouse model of Parkinson's disease. Pharmacol Res. (2022) 177:106133. doi: 10.1016/j.phrs.2022.106133 35182746

[56] Xia M Zhang Y Wu H Zhang Q Liu Q Li G . Forsythoside B attenuates neuro-inflammation and neuronal apoptosis by inhibition of NF-κB and p38-MAPK signaling pathways through activating Nrf2 post spinal cord injury. Int Immunopharmacol. (2022) 111:109120. doi: 10.1016/j.intimp.2022.109120 35944463

[57] Zhang F Lv T Li J Lian J Wu H Jin Y . Citrate synthase lysine K215 hypoacetylation contributes to microglial citrate accumulation and pro-inflammatory functions after traumatic brain injury. CNS Neurosci Ther. (2024) 30:e14567. doi: 10.1111/cns.14567 38421106 PMC10851320

[58] Lopez-Rodriguez AB Hennessy E Murray CL Nazmi A Delaney HJ Healy D . Acute systemic inflammation exacerbates neuroinflammation in Alzheimer's disease: IL-1β drives amplified responses in primed astrocytes and neuronal network dysfunction. Alzheimers Dement. (2021) 17:1735–55. doi: 10.1002/alz.12341 34080771 PMC8874214

[59] Bialuk I Jakubów P Winnicka MM . Significance of IL-6 deficiency in recognition memory in young adult and aged mice. Behav Genet. (2019) 49:415–23. doi: 10.1007/s10519-019-09959-6 31129771 PMC6554246

[60] Chen LL Zhang DR Li J Wang HM Song CH Tang X . Albicanol alleviates D-galactose-induced aging and improves behavioral ability via by alleviating oxidative stress-induced damage. Neurochem Res. (2021) 46:1058–67. doi: 10.1007/s11064-020-03220-x 33761044

[61] Yang R Zhu F Mo W Li H Zhu D He Z . A new plant active polysaccharide from Nicotiana improves the lead-led impairment of spatial memory in mice by modulating the gut microbiota and IL-6. Foods. (2024) 13(19):3177. doi: 10.3390/foods13193177 39410212 PMC11475524

[62] Jo JH Park EJ Lee JK Jung MW Lee CJ . Lipopolysaccharide inhibits induction of long-term potentiation and depression in the rat hippocampal CA1 area. Eur J Pharmacol. (2001) 422:69–76. doi: 10.1016/s0014-2999(01)01075-5 11430915

